# Supplementary material for: Canalized Morphogenesis Driven by Inherited Tissue Asymmetries in Hydra Regeneration
Source: Genes (Basel). 2022 Feb 16;13(2):360. doi: 10.3390/genes13020360 (PMC8872179; doi:10.3390/genes13020360)
Supplement: Supplementary file 1 [file genes-13-00360-s001.zip › genes-1523290_SuppIymentary figures.pdf]

Supplementary Information

Supplementary Figures

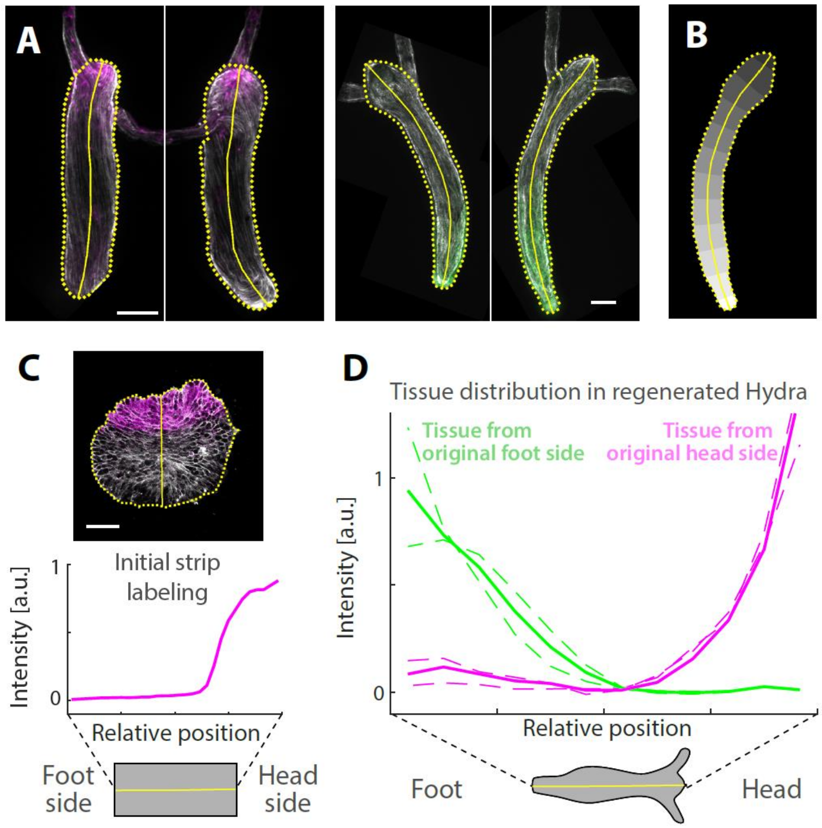

Figure S1. Analysis of the tissue redistribution during regeneration. The distribution of tissue originating from tissue strips labeled either at their head-facing side (magenta) or foot-facing side (green) is determined from the relative labeling intensity in regenerated animals. (A) Spinning disk confocal mean projections of the fluorescence signal of uncaged Abberior CAGE 552 for animals regenerating from a strip labeled at its head side (Left, magenta) or its foot side (Right, green). For each regenerating animal, a pair of images are acquired by flipping the sample to visualize the tissue distribution on both sides. (B) A schematic depiction of the definition of the segmented regions used for measuring the position-dependent label intensity along the body axis (Methods). The outline of the animal's body without the tentacles (dashed yellow line) and the centerline along the body axis (yellow line) are indicated. (C) Top: Scanning confocal images of an excised tissue strip labeled at one end by laser-induced uncaging of Abberior CAGE 552. Bottom: Graph showing the relative label intensity along the excised tissue strip. The images In (A,C) show an overlay of the uncaged label intensity (magenta/green) and the lifeact-GFP signal (gray). (D) Graph showing the relative label intensity along the body axis for the two animals shown in (A), regenerating from a tissue strip labeled on its head side (magenta) or foot side (green), respectively. For each animal, the distributions from

either side are shown (dashed lines) together with the average distribution from both sides (thick lines).

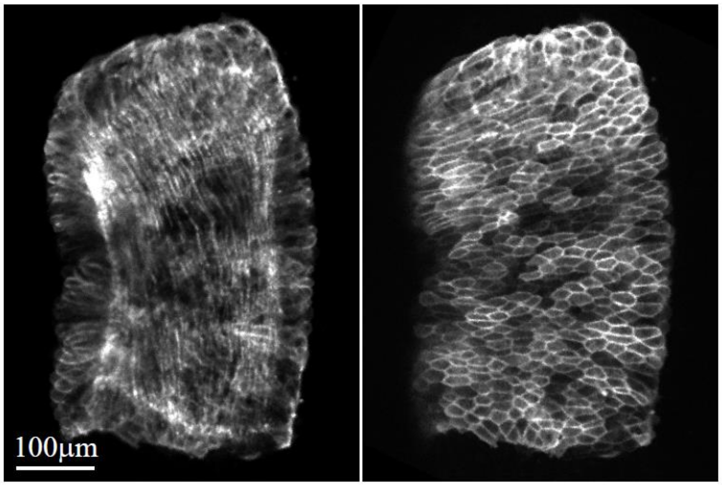

Figure S2. Size and shape of a rectangular tissue strip. Spinning disk confocal projected images of the basal (Left) and apical (Right) surfaces of a rectangular tissue strip excised from the gastric region of a mature *Hydra* expressing lifeact-GFP in the ectoderm. The supracellular actin fibers that are parallel to the body axis of the parent animal are visible in the basal surface of the ectoderm (Left) and the cellular cortices are visible in the apical surface (Right). The length and width of the strips (parallel and perpendicular to the body axis of the parent animal, respectively) is measured by counting cells parallel and perpendicular to the body axis of the parent animal (the size in terms of cell numbers is more reliable since the excised tissue often contracts generating large changes in the apparent size and width of the samples). The measured length and width for a population of excised strips are 36±8 and 16±4 cells (mean±standard deviation, N=11), respectively, for a total of ~600 ectodermal cells.

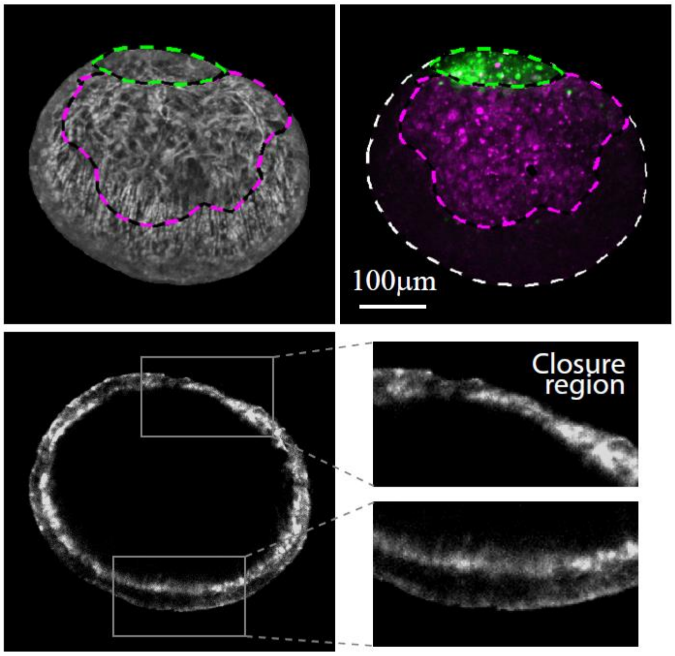

Figure S3. The shape of the initial folded spheroid. Images of a folded spheroid formed from a rectangular tissue strip differentially labeled at its head-facing side with uncaged Abberior CAGE 552 (magenta) and its foot-facing side with uncaged Abberior CAGE 635 (green) 3 hours after excision. Top: Projected spinning-disk confocal images of the spheroid imaged from the side, so that the closure region appears in the upper right region of the image. An overlay of the fluorescent markers labeling the head (magenta) and foot-facing (green) sides of the strip are shown (Right), together with an image of the supracellular actin fibers labeled with lifeact-GFP (gray) on the basal surface of the ectoderm overlaid with contours depicting the labeled regions (Left). The actin fibers appear disordered in the closure region (upper half of the spheroid), while the parallel organization of the fibers is maintained away from the closure region (lower half of the spheroid). Bottom: A spinning disk confocal medial cross-section of the tissue spheroid, showing the thickness of the ectodermal layer. The layer becomes thinner in the closure region, due to the tissue stretching that occurs during the folding process, as the tissue seals into a hollow spheroid.

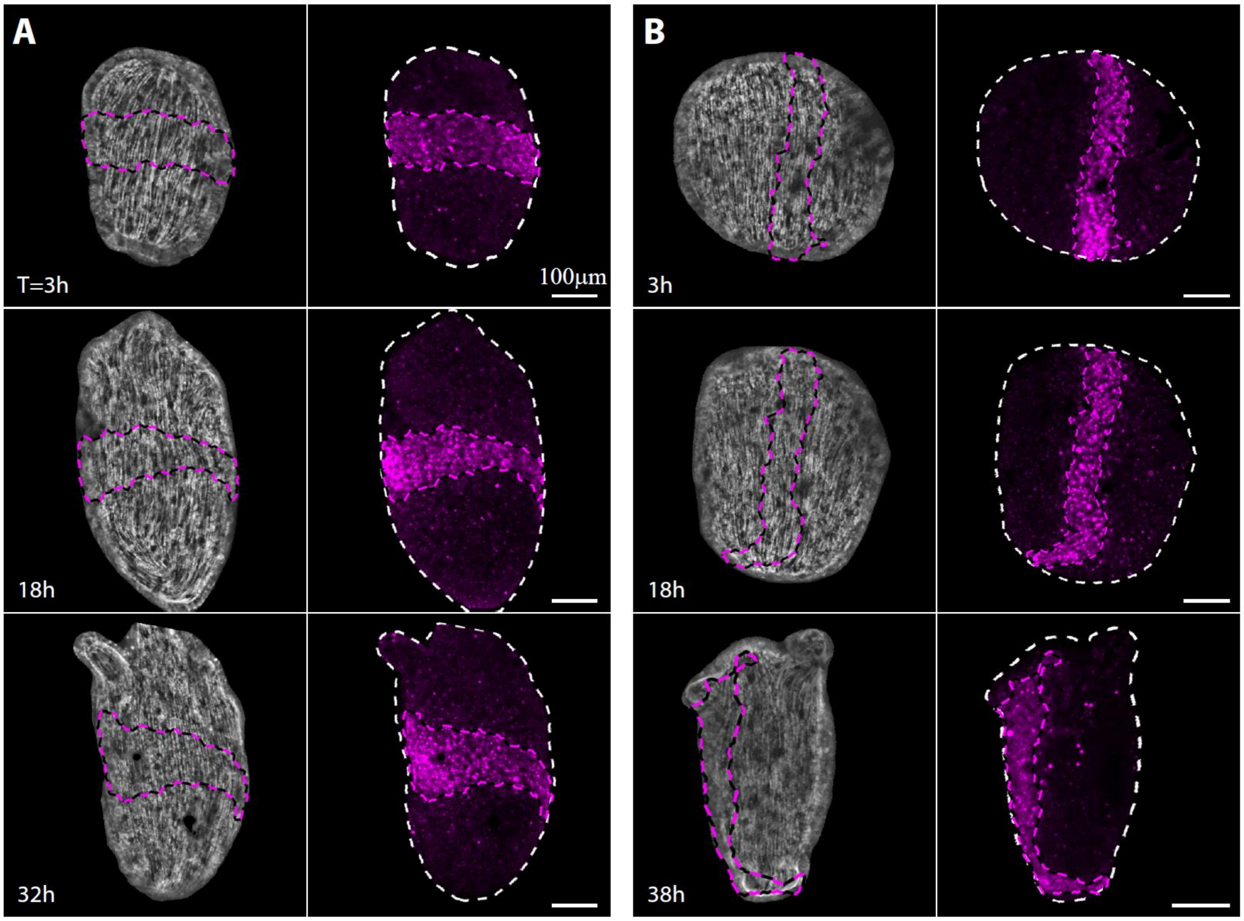

Figure S4. Tissue dynamics and actin fiber organization during regeneration of strips labeled along a medial or central line. Images from time-lapse movies of regenerating tissue strips labeled by laser-induced uncaging immediately following excision along a medial line perpendicular to the body axis of the parent animal (A) or a central line parallel to the body axis of the parent animal (B) (Movie 1). Left: Projected spinning disk confocal images of the supracellular actin fibers in the basal surface of the ectoderm (lifeact-GFP, gray), overlaid with contours of the labeled tissue (magenta). Right: Projected spinning disk confocal images of the fluorescent marker in the labeled tissue originating from the central line in the middle of the strip (uncaged Abberior CAGE 552; magenta). The white dashed line marks the tissue outline.

Supplementary Movies

Movie S1. Tissue dynamics in regenerating tissue strips labeled along the central line. Time-lapse movies depicting the tissue dynamics in regenerating tissue strips expressing lifeact-GFP and labeled along a central line, parallel to the body axis of the parent animal. Upper and lower panels depict two different samples showing the tissue dynamics during regeneration from different viewpoints. The labeled line remains parallel to the ectodermal actin fibers which are aligned with the regenerated animal's body axis. Left: Projected spinning disk confocal images of the supracellular actin fibers in the basal surface of the ectoderm (lifeact-GFP, gray), overlaid with contours of the labeled tissue (magenta). Right: Projected spinning disk confocal images of the fluorescent marker in the labeled tissue originating from the central line in the middle of the strip (uncaged Abberior CAGE 552; magenta). The white dashed line marks the tissue outline. The elapsed time from excision is displayed (hrs:min), and the scale bar is 100 µm.

Movie S2. Tissue dynamics in a regenerating strip viewed from its back side. Time-lapse movie depicting the tissue dynamics in a regenerating tissue strip expressing lifeact-GFP in the ectoderm and differentially labeled at its head and foot-facing sides. The sample is oriented so that the back side of the tissue (opposite from the initial closure region) is visible. Projected spinning disk confocal images of the basal (Left) and apical (Middle) surfaces of the ectodermal layer are shown (lifeact-GFP, gray), overlaid with contours depicting the tissue regions originating from the head (magenta) and foot (green) facing sides of the strip. The supracellular actin fibers are visible in the basal surface of the ectoderm (Left) and the cellular cortices are visible in the apical surface (Middle). Right: Projected spinning disk confocal images of the fluorescent markers labeling the originally head (uncaged Abberior cage 635; magenta) and foot-facing (uncaged Abberior CAGE 552; green) sides of the strip. The outline of the tissue is also denoted (white dashed line). The elapsed time from excision is displayed (hrs:min), and the scale bar is 100 µm.

Movie S3. Tissue dynamics in a regenerating strip viewed from its front side. Time-lapse movie depicting the tissue dynamics in a regenerating tissue strip expressing lifeact-GFP in the ectoderm and differentially labeled at its head and foot-facing sides. The sample is oriented so that the initial closure region is visible, showing tissue from the head-facing edge of the strip (magenta) adhered to tissue from the foot-facing edge (green). Projected spinning disk confocal images of the basal (Left) and apical (Middle) surfaces of the ectodermal layer are shown (lifeact-GFP, gray), overlaid with contours depicting the tissue regions originating from the head (magenta) and foot (green) facing sides of the strip. The supracellular actin fibers are visible in the basal surface (Left) and the cellular cortices are visible in the apical surface (Middle). Right: Projected spinning disk confocal images of the fluorescent markers labeling the originally head (uncaged Abberior CAGE 552; magenta) and foot-facing (uncaged Abberior CAGE 635; green)

sides of the strip. The outline of the tissue is also denoted (white dashed line). The elapsed time from excision is displayed (hrs:min), and the scale bar is 100 µm.

Movie S4. Actin fiber reorganization in the head region of a regenerating tissue strip. Time-lapse movie depicting the tissue dynamics and cytoskeletal organization in a regenerating tissue strip expressing lifeact-GFP in the ectoderm and differentially labeled at its head and foot-facing sides. Left: Projected spinning disk confocal images of the fluorescent markers labeling the originally head (uncaged Abberior CAGE 552; magenta) and foot-facing (uncaged Abberior CAGE 635; green) sides of the strip. The outline of the tissue is also denoted (white dashed line). Right: Projected spinning disk confocal images of the supracellular actin fibers in the basal surface of the ectoderm (lifeact-GFP, gray) overlaid with contours depicting the tissue regions originating from the head (magenta) and foot (green) facing sides of the strip. The location of the aster-like defect in the organization of the supracellular actin fibers is denoted by an orange box. The defect forms within the tissue originating from the head-facing side of the strip (magenta), at a site that coincides with the location of the mouth of the regenerated animal. The elapsed time from excision is displayed (hrs:min), and the scale bar is 100 µm.

Movie S5. Actin fiber reorganization in the foot region of a regenerating tissue strip. Time-lapse movie depicting the tissue dynamics and cytoskeletal organization in a regenerating tissue strip expressing lifeact-GFP in the ectoderm and differentially labeled at its head and foot-facing sides. Left: Projected spinning disk confocal images of the fluorescent markers labeling the originally head (uncaged Abberior CAGE 552; magenta) and foot-facing (uncaged Abberior CAGE 635; green) sides of the strip. The outline of the tissue is also denoted (white dashed line). Right: Projected spinning disk confocal images of the supracellular actin fibers in the basal surface of the ectoderm (lifeact-GFP, gray) overlaid with contours depicting the tissue regions originating from the head (magenta) and foot (green) facing sides of the strip. The location of the two horseshoe-like defects in the organization of the supracellular actin fibers are denoted by cyan boxes (different dashed lines are used to discriminate between the two defects). The defects form within the tissue originating from the foot-facing side of the strip (green), and eventually merge in the region that becomes the foot of the regenerated animal. The elapsed time from excision is displayed (hrs:min), and the scale bar is 100 µm.
